# PAI-1 and functional blockade of SNAI1 in breast cancer cell migration

**DOI:** 10.1186/bcr2203

**Published:** 2008-12-03

**Authors:** Elizabeth Fabre-Guillevin, Michel Malo, Amandine Cartier-Michaud, Hector Peinado, Gema Moreno-Bueno, Benoît Vallée, Daniel A Lawrence, José Palacios, Amparo Cano, Georgia Barlovatz-Meimon, Cécile Charrière-Bertrand

**Affiliations:** 1 DYNAMIC Team, IBISC FRE 3190 CNRS – Université d'Evry Val d'Essonne, Genopole, Evry 91000, France; 2 Department of Medical Oncology, Hopital Européen Georges Pompidou, Paris cedex 15 75908, France; 3 Departamento de Bioquímica, Instituto de Investigaciones Biomedicas 'Alberto Sols', (CSIC-UAM), Madrid 28029, Spain; 4 Molecular Pathology Program, Centro Nacional de Investigaciones Oncologicas (CNIO), Madrid 28029, Spain; 5 Department of Internal Medicine, University of Michigan School of Medicine, Ann Arbor MI 48109-0644, USA; 6 University Paris 12 – Val de Marne, Créteil cedex 94010, France

## Abstract

**Introduction:**

Snail, a family of transcriptional repressors implicated in cell movement, has been correlated with tumour invasion. The Plasminogen Activation (PA) system, including urokinase plasminogen activator (uPA), its receptor and its inhibitor, plasminogen activator inhibitor type 1(PAI-1), also plays a key role in cancer invasion and metastasis, either through proteolytic degradation or by non-proteolytic modulation of cell adhesion and migration. Thus, Snail and the PA system are both over-expressed in cancer and influence this process. In this study we aimed to determine if the activity of SNAI1 (a member of the Snail family) is correlated with expression of the PA system components and how this correlation can influence tumoural cell migration.

**Methods:**

We compared the invasive breast cancer cell-line MDA-MB-231 expressing SNAI1 (MDA-mock) with its derived clone expressing a dominant-negative form of SNAI1 (SNAI1-DN). Expression of PA system mRNAs was analysed by cDNA microarrays and real-time quantitative RT-PCR. Wound healing assays were used to determine cell migration. PAI-1 distribution was assessed by immunostaining.

**Results:**

We demonstrated by both cDNA microarrays and real-time quantitative RT-PCR that the functional blockade of SNAI1 induces a significant decrease of PAI-1 and uPA transcripts. After performing an *in vitro *wound-healing assay, we observed that SNAI1-DN cells migrate more slowly than MDA-mock cells and in a more collective manner. The blockade of SNAI1 activity resulted in the redistribution of PAI-1 in SNAI1-DN cells decorating large lamellipodia, which are commonly found structures in these cells.

**Conclusions:**

In the absence of functional SNAI1, the expression of PAI-1 transcripts is decreased, although the protein is redistributed at the leading edge of migrating cells in a manner comparable with that seen in normal epithelial cells.

## Introduction

Epithelial-mesenchymal transition (EMT) is a process whereby epithelial cell layers lose polarity and cell-cell contacts and undergo a dramatic remodelling of the cytoskeleton. EMT is characterised by a loss of intercellular adhesion, down-regulation of epithelial markers, up-regulation of mesenchymal markers, and acquisition of a spindle-shape and single-cell migration [1,2]. Many of the molecular changes occurring during developmental EMT are also characteristics of most aggressive metastatic cancer cells [2-5].

Important in EMT is the Snail family of transcriptional repressors whose members including SNAI1, also known as snail, and SNAI2, also known as slug [6]. One of the major effects of Snail family molecules is the induction of a mesenchymal and invasive phenotype [7]. This process includes alterations in the expression of a wide number of proteins involved in cell-to-cell and cell-to-extracellular matrix interactions, as well as cytoskeletal reorganisation and migration [7,8]. When over-expressed in epithelial Madin Darby Canin Kidney (MDCK) cells, SNAI1 induces a full EMT leading to the acquisition of a motile, invasive phenotype [9,10]. In agreement with this role, SNAIl has also been found to down-regulate the expression of epithelial genes, including E-cadherin [11-14] and to induce the expression of mesenchymal genes [15,16]. Conversely, Olmeda and colleagues demonstrated that SNAI1 silencing by stable RNA interference in MDCK-SNAI1 cells induced a complete mesenchymal to epithelial transition (MET), associated with the up-regulation of E-cadherin and down-regulation of mesenchymal markers [17].

In several tumours, including breast cancers, SNAI1 has been correlated with invasive growth potential, partly because of its ability to directly repress transcription of genes whose products are involved in cell-cell adhesion [11,15,18,19]. Several studies have shown that SNAI1 is found in the invasive regions of tumours [15,20,21]. Moreover, Blanco and colleagues [18] have reported that SNAI1 expression is correlated with both histological grade and lymph node extension in breast cancers.

It has also been established that plasminogen activator inhibitor-1 (PAI-1), urokinase plasminogen activator (uPA) and uPA receptor (uPAR), members of the plasminogen activation system (PA system), play a key role in cancer invasion and metastasis [22,23]. PAI-1 is over-expressed in the immediate vicinity of tumours [24], and preferentially localised to the stromal area [25]. In addition to catalysing the degradation of the extracellular matrix (ECM) and modulating cell adhesion [26], the PA system enhances both cell proliferation [27] and migration [28-34]. Consistent with their role in cancer dissemination, high levels of uPA, PAI-1 and uPAR correlate with adverse patient outcome [35-37]. In particular, the prognostic value of PAI-1 has recently been validated in breast cancer patients [38]. PAI-1 may represent a key molecule in the rapid attachment/detachment events required for cell migration, by its ability to both decrease its affinity for vitronectin in the ECM and to increase its affinity for endocytic receptors such as the lipoprotein receptor-related protein (LRP) in response to PA binding [33,39-43]. It has also been demonstrated that PAI-1 can induce cell behaviour changes, such as proliferation of cancer cells, indirectly through cell signalling pathways [44]. Thus, PAI-1 can modulate cell adhesion and migration via direct interactions with integrins, LRP, uPA-uPAR and with the ECM [43,45,46], or through indirect classical signalling pathways.

Both SNAI1 and PA system components play a major role in tumour migration and have been localised to the tumour leading edge [15,20,21,24,25]. Moreover, three recently published studies directly or indirectly underline a link between the over-expression of SNAI1 and the PA system. In the first [47], a dichotomous role for the transcription factor AFT3 in cancer is demonstrated. This factor up-regulates the expression of PAI-1, uPA and SNAI1 in breast cancer cells. The second article [48] shows, using colon cancer cells, that the induction of SNAI1 expression represses diverse genes, including uPAR, involved in epithelial differentiation, metabolism and signalling. In the third paper, it has been shown that over-expression of SNAI1 and SNAI2 in canine kidney epithelial cells leads to over-expression of PAI-1 [9].

In the present study, a 'reverse' analysis was used in breast cancer cells where the activity of SNAI1 had been abolished, with the aim of examining a possible link between SNAI1 and PAI-1 during EMT, a process occurring during tumour progression.

Our data show that functional blockade of SNAI1 (SNAI1-dominant negative (DN)) leads to a partial re-expression of E-cadherin, and induces differential expression of EMT-related genes. This is confirmed by RT-PCR of PA system genes, where PAI-1 and uPA are decreased. In SNAI1-DN cells, cell migration is quantitatively decreased, and this is associated with morphological changes with SNAI1-DN cells displaying a more collective behaviour compared with MDA-mock cells that tend to migrate individually. PAI-1 is differently distributed in SNAI1-DN cells, with numerous lamellipodia decorated with anti-PAI-1 antibodies, compared with MDA-mock cells that demonstrate a more homogeneous distribution of PAI-1.

## Materials and methods

### Chemicals and reagents for cell culture

Leibovitz's L-15 Medium with GlutaMAX, penicillin-streptomycin, trypsin-EDTA and PBS were all purchased from GIBCO^® ^Invitrogen Cell Culture (Paisley, UK). FCS was purchased from Bio Media (Quebec, Canada). BSA was purchased from Sigma (Saint Louis, USA).

### Cells and cell culture

Three established cell lines were used in this study: MDA-MB-231, an invasive breast cancer cell line, and two derived clones obtained after stable transfection with either an empty pcDNA3 plasmid (clone MDA-mock) or with a dominant negative (DN) construct of SNAI1, pcDNA3-DN-SNAI1 lacking the N-terminal SNAG domain (clone SNAI1-DN) [10].

The plasmid pcDNA3 contains the cytomegalovirus (CMV) promoter that induces high-level constitutive expression in a variety of mammalian cell lines, notably in epithelial and endothelial cells. The high quantity of SNAI1-DN protein produced by the pcDNA3 vector should therefore be sufficient to overcome the short half-life of this protein.

Cells were grown in Leibovitz's L-15 Medium with GlutaMAX supplemented with 10% FCS, 100 U/mL penicillin and 100 U/mL streptomycin. Cultures were selected with 400 μg/mL G418 to generate stable cell lines and maintained in exponential growth in a humidified atmosphere at 37°C without carbon dioxide. Cells were fed every third day and only used at passage four to seven for the experiments. All experiments were performed three times on each of the three cell lines cultivated in usual conditions (three days in Leibovitz's L-15 Medium completed with FCS).

### Cell staining and immunocytochemistry

4', 6-diamidino-2-phenylindole (DAPI), dihydrochloride Alexa Fluor 568 phalloïdin and Alexa Fluor 488 secondary antibodies were used in this study (Invitrogen – Molecular Probe – Fischer Bioblock Scientific, Illkirch, France).

For F-actin and DAPI staining, culture medium was aspirated and cells washed three times with PBS^+/+ ^and fixed by incubation with prepared formaldehyde 3.7% in PBS^-/- ^(pH 7.4) for 10 minutes at room temperature. Cells were again washed three times in PBS^-/- ^and permeabilised for three minutes with Triton 0.1%. After three washings, specific sites were saturated with 1% BSA in PBS^-/- ^for 20 minutes, and incubated with 5 U/mL Alexa Fluor 568 phalloïdin for 30 minutes. Cells were washed three times with PBS^-/- ^and incubated with 300 nM DAPI for four minutes. After PBS^-/- ^washing, fixed stained cells were stored in PBS^-/- ^at 4°C before immunostaining.

For immunostaining, cells were incubated with primary antibodies: antibodies anti-PAI-1 (1:50) (polyclonal rabbit anti-PAI-1 H-135 primary antibody, Santa Cruz Biotechnology; Santa Cruz, CA, USA); antibody anti E-cadherin (1:200) (monoclonal mouse anti E-cadherin; BD Biosciences, le Pont de Claix, France), during two hours.

Primary antibodies were revealed (one hour incubation) with Alexa Fluor 488 secondary antibodies (anti-rabbit IgG 1:200; anti-mouse IgG 1:200). Stained cells were mounted under coverslips in Mowiol buffer.

Samples were examined using a 40×/1.30 Plan-Neofluar oil immersion objective lens on an Apo Tome Axiovert 200 (Zeiss, Le Pecq, France). All images were analysed using AxioVision 4.0 (Zeiss, Le Pecq, France) and figures were assembled using Adobe Photoshop 6.0 (Adobe System, Paris, France).

### Microarray analysis

Total RNA from the different cell lines was extracted using Trizol reagent (Life Technologies Inc., Gaithersburg, MD, USA) as indicated by the manufacturer. To synthesise the double-stranded cDNA, 3 μg of total RNA using T7-(dThd) 24 oligo primers by the Superscript Choice System (Life Technologies Inc., Gaithersburg, MD, USA) was used. *In vitro *transcription was conducted with Megascript T7 (Ambion, Austin, TX, USA). Amplified RNA was obtained and purified using Trizol reagent, and 3 μg of amplified RNA was used to generate fluorescence antisense RNAs by transcriptional synthesis using the SuperScript enzyme protocol (Life Technologies Inc., Gaithersburg, MD). All of the samples were labelled with either Cy5-dUTP or Cy3-dUTP fluorochromes (Amersham, Uppsala, Sweden). Hybridisation was performed into the 'CNIO Oncochip' cDNA microarray v 1.1c (CNIO Genomic Unit, Madrid, Spain) as previously described [49]. The microarray platform contains 9726 clones corresponding to 6386 different genes, which includes 2489 clones that have been printed in duplicate to assess reproducibility. Duplicate samples from the SNAI1-DN transfectant were labelled with dUTP-Cy5 and hybridised against the dUTP-Cy3-labelled MDA-mock controls. Two hybridisations were performed. Slides were washed, dried and then scanned in a Scanarray 5000 XL scanner (GSI Lumonics, Kanata, Ontario, Canada) to obtain 10 μm resolution images, which were then quantified using the GenePix Pro 5.0 program (Axon Instruments Inc., Union City, CA). Data from the fluorescence intensity measurements of each array experiment were processed using GenePix Pro 5.0 and Microsoft Excel programs, as described before [49]. For statistical analysis, we selected genes with expressions that differed by a factor of at least two-fold with respect to the MDA-mock control cells.

### RNA extraction and real-time quantitative RT-PCR

Total RNA was isolated from cell cultures as previously described by Chomczynski and colleagues [50]. RNA quantity and RNA purity were performed by spectrophotometry using GeneQuant II DNA/RNA calculator (Pharmacia Biotech, Piscataway, N.J, USA). RNA integrity was checked by electrophoresis performed on agarose gel. Samples were then treated by DNase I (Invitrogen, Cergy Pontoise, France) in order to remove contaminating genomic DNA. An amount of 1 μg of total RNA was reverse-transcribed using random hexamers as primers and Superscript II reverse transcriptase (Invitrogen, Cergy Pontoise, France). Real-time quantitative RT-PCR of the obtained cDNA was performed in triplicate on an ABI Prism 7000 Sequence Detection System (Applied Biosystems, Courtaboeuf, France).

In these conditions, the expression of mRNAs encoding for PAI-1, uPA and uPAR was quantified using assay-on-demand TaqMan MGB probes Hs00170182_m1 (uPA), Hs00182181_m1 (uPAR), Hs00167155_m1 (PAI-1) predeveloped from Applied Biosystems (Courtaboeuf, France), labelled with FAM reporter dye and a non-fluorescent quencher. We used 18S RNA as control to normalise gene expression using assay-on-demand TaqMan MGB probe Hs99999901_s1 (Applied Biosystems, Courtaboeuf, France). Then, 5 ng cDNA was used as template for real-time PCR. The thermal cycler parameters for the real-time PCR were two minutes at 50°C (incubation with AmpErase UNG), 10 minutes at 95°C (activation of Taq polymerase) followed by 40 cycles with 15 seconds at 95°C and one minute at 60°C. All of these steps (with associated products) were performed according to the recommendations of the manufacturer.

The relative expression level of the genes was calculated by measuring the threshold cycle (C_T_) (using version 1.1 software from the ABI Prism 7000 Sequence Detection System) and use of the comparative C_T _method as described by the provider (ABI Prism user bulletin #2). Levels of transcripts in the different clones (MDA-mock, SNAI1-DN) were determined in reference to those obtained for the parental MDA-MB-231 cell line cultured in FCS (the latter being standardised to one).

Semi-quantitative RT-PCR analysis for detection of E-cadherin and glyceraldehyde-3-phosphate dehydrogenase was also performed, using appropriate primers as previously described [51].

### *In vitro *wound healing assay

*In vitro *wound-healing assay was performed as previously described [15,52]. MDA-mock and SNAI1-DN cells were seeded on tissue culture dishes. Cell monolayers were then gently scratched with a pipette tip across the entire diameter of the dish and extensively rinsed with medium to remove all cellular debris. The area of denuded surface (500 to 700 μm in width) was quantified immediately after wounding and again three and five hours later. The extent of wound closure was determined by calculating the ratio between the surface area of the wound for each time point and the surface of the initial wound. These data were then expressed as the percentage of wound closure relative to the control conditions for each experiment. The dish was placed under an Axiovert 200 inverted microscope (Zeiss, Le Pecq, France) and images were obtained using a high-resolution microscopic camera Axiocam MRm vers.3 FireWire (D) camera (Zeiss, Le Pecq, France) connected to the microscope. Phase-contrast pictures were analysed by AxioVision 4.0 software (Zeiss, Le Pecq, France).

### Statistical analysis

The data were analysed using a Student's t-test (GraphPad Software Inc., San Diego, CA, USA), and p < 0.05 were considered to indicate significant differences.

## Results and discussion

### SNAI1 functional blockade and E-cadherin expression

In order to study the possible link between SNAI1 and the PA system during tumour progression, the MDA-MB-231 cell line, an invasive breast cancer cell line was used to generate stable cell lines transfected with a dominant-negative construct of SNAI1 lacking the N-terminal transactivation domain SNAG [8]. This dominant-negative version is able to interact with the same target sequences as endogeneous SNAI1, thus blocking SNAI1 function when it is over-expressed.

**Figure 1:**
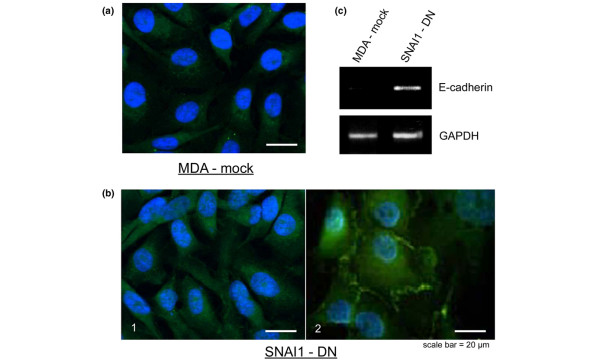
**Immunofluorescence analysis of E-cadherin (green) in MDA-mock and SNAI1-DN cells. Nuclei are visualised by 4', 6-diamidino-2-phenylindole (DAPI) stain**. **(a) **The MDA-mock control cell line transfected with a control vector (mock) presents no cell-cell contact, and the immunostaining of E-cadherin is very low and diffused. **(b_1_) **SNAI1-DN cells have acquired an epithelial like phenotype with E-cadherin stronger expression **(b_2_) **sometimes organised specifically at cell-cell contacts. **(c) **Semi-quantitative RT-PCR analysis of E-cadherin mRNA in MDA-mock and SNAI1-DN cells. GADPH mRNA levels were detected as a control of cDNA inputs. SNAI1-DN expression in MDA-MB-231 cells lead to the re-expression of E-cadherin mRNA.

As shown in Figure 1a, the MDA control cell line transfected with the control vector (mock) maintained the same non-epithelial phenotype as parental MDA-MB-231 cells, and showed few cell-cell contacts. Performing semi-quantitative RT-PCR analysis with E-cadherin, we demonstrated that SNAI1 functional blockade lead to the re-expression of E-cadherin mRNA in MDA-MB-231 cells (Figure 1c). However, E-cadherin immunostaining is not clearly found at cell-cell contacts (Figure 1b), as would be expected in an epithelial phenotype, and western blot did not show any 120 kD protein (data not shown). This absence of E-cadherin protein in SNAI1-DN cells suggests that E-cadherin degradation still occurs when we abolish the effect of SNAIl in our cancer cells. Indeed, in addition to transcriptional control, considered to be the major mechanism regulating E-cadherin expression, recent data also suggest that post-translational mechanisms, such as ubiquitination, endocytosis and lysosomal degradation of E-cadherin, occur during EMT, and are important for both embryological development and tumour progression [53,54].

These observations are consistent with recently published work using MDA-MB-231 cell lines stably transfected by short hairpin RNA directed against SNAI1 to knock-down SNAI expression [55]. These studies showed an increase of E-cadherin mRNA level but no E-cadherin protein expression and a decrease in the expression of the mesenchymal markers, fibronectin and vimentin, suggesting that SNAI1 silencing induces a partial reversion of EMT in MDA-MB-231. Moreover, Janda and colleagues indicate that the early stages of EMT involve post-translational down-regulation of E-cadherin, whereas loss of E-cadherin via transcriptional repression is a late event in EMT [56]. In the reversion of EMT (i.e. MET), we can hypothesise that a reverse situation would be observed: expression of E-cadherin first at transcriptional level and then at translational and post-translational levels.

To further highlight the relation between SNAI1 and the PA system in EMT occuring during tumour progression, we used cDNA microarrays to compare MDA-mock and SNAI1-DN cell by differential gene expression.

### SNAI1 functional blockade leads to PAI-1 down-regulation

Of 6386 genes compared (microarray accession number [GSE:10395] deposited in the Gene Expression Omnibus), a total of 99 genes were found to be differentially expressed in response to SNAI1 functional blockade [see Additional data file 1]. As shown in Table 1, nine of the 99 target genes differentially expressed in SNAI1-DN clone are specifically implicated in EMT and in matrix remodelling, these include several members of the PA system. Of particular note, the expression of PAI-1 is decreased 7.2-fold in the SNAI1-DN clone (Table 1), compared with the MDA-mock clone.

**Table 1:** Epithelial to mesenchymal transition (EMT) related genes modified in a clone expressing a dominant-negative form of SNAI1 (SNAI1-DN) in comparison with MDA-mock control cells

GenBank number	SNAI1-DN	Description
**EMT related (n = 9)**		
		
[Genbank:AA447737]	-2.12	*CALD1*, caldesmon 1
[Genbank:AA284668]	-2.45	*PLAU*, plasminogen activator, urokinase
[Genbank:AA136707]	-2.12	*PLOD2*, procollagen-lysine, 2-oxoglutarate 5-dioxygenase (lysine hydroxylase) 2
[Genbank:N75719]	-7.22	*SERPINE1*, serine (or cysteine) proteinase inhibitor, clade E (nexin, plasminogen activator inhibitor type 1), member 1
[Genbank:H96738]	2.29	*CDH11*, cadherin 11, type 2, OB-cadherin (osteoblast)
[Genbank:H15267]	2.48	*CHL1*, cell adhesion molecule with homology to L1CAM (close homologue of L1)
[Genbank:AA598794]	3.87	*CTGF*, connective tissue growth factor
[Genbank:W51794]	3.49	*MMP3*, matrix metalloproteinase 3 (stromelysin 1, progelatinase)
[Genbank:AA479202]	2.58	*TIMP3*, tissue inhibitor of metalloproteinase 3 (Sorsby fundus dystrophy, pseudo-inflammatory)

Nine of the 99 target genes differentially expressed in SNAI1-DN clone are implicated in the EMT process and matrix remodelling, including some members of the plasminogen activation system. Particularly, the expression of PAI-1 is decreased by 7.2-fold in SNAI1-DN clones. - = down-regulation; + = up-regulation.

**Figure 2:**
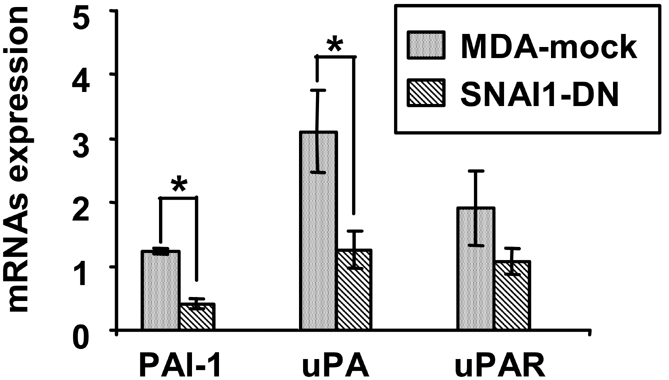
**Expression of the Plasminogen Activation system (PA) system components mRNAs (Plasminogen Activator Inhibitor type-1 (PAI-1), urokinase Plasminogen Activator (uPA), urokinase Plasminogen Activator Receptor (uPAR)) in SNAI1-DN versus MDA-mock cells**. Steady state levels of the PA system component mRNAs were evaluated by real-time quantitative RT-PCR analysis of total RNA of SNAI1-DN and MDA-mock cells cultured as described. These levels are determined in reference to those obtained for the original MDA-MB-231 cell line which were standardised to one. Mean values (standard error of the mean) of four independent cultures are shown.* p < 0.05.

These data are similar to those obtained [9] that demonstrated that PAI-1 was the most affected gene after over-expression of SNAI1. These data are also in agreement with several studies showing that PA system components are involved in tumour evolution [22,23] including tumoural transitions EMT and mesenchymal to amoeboid transition (MAT)) [57-61]. Therefore, we focused on PA system genes and confirmed the microarray results by quantitative real-time RT-PCR. Both levels of PAI-1 and uPA transcripts were strongly decreased in SNAI1-DN cells compared with MDA-mock cells, although uPAR transcripts remained unchanged (Figure 2).

These results from more precise and sensitive methods, confirmed the microarray data and showed that the expression of PA system component transcripts, especially PAI-1 and uPA, are dependent on SNAI1 functionality.

This relation between SNAI1 and the PA system is consistent with the observations of Kuphal and colleagues [61] and Hou and colleagues [62]. Kuphal and colleagues, using melanoma cells, pointed out that the loss of E-cadherin leads to the up-regulation of NFkB activity, and that after re-expression of E-cadherin, NFkB activity was down-regulated [62]; while Hou and colleagues demonstrated that NFkB activity independently induces the expression of PAI-1 [63]. We therefore hypothesise that in the SNAI1-DN cells the functional deficiency of SNAI1 leads to the re-expression of E-cadherin (Figure 1), which in turn down-regulates NFkB leading to the decreased expression of PAI-1 mRNA.

SNAI1 is also known to act as a transcriptional repressor through binding to E-boxes [8]. It is possible that functional blockade of SNAI1 could have lead to an increase in PAI-1 expression rather than a decrease (Figure 2). However, in this context, it is worth noting that Allen and colleagues suggested that the E-box motif could function as a 'platform' for recruitment of either positive and negative regulators of PAI-1 expression [64]. This implies that if the E-box is indeed the pathway of SNAI1 influence, then its effect on PAI-1 transcription cannot be easily anticipated. And this effect depends "on the stimulus and/or the growth state" [64]. Moreover, Munshi and colleagues showed that *de novo *E-cadherin mediates adhesive contacts and leads to a decrease of the global PAI-1 levels [65]. Whereas in the opposite situation – blockade of E-cadherin-dependant cell-cell adhesion – a stimulation of uPA expression was observed [66,67]. Consistent with these latter studies, our data show a decrease of uPA transcript in SNAI1-DN cells.

As numerous papers have already described, mechanisms of cancer migration are dependant on uPA, and as PAI-1 transcripts were the most affected by the changes in SNAI1 expression (over-expression or functional blockade), we focused on the link between SNAI1 and PAI 1. Moreover, two indirect arguments suggest a potential link between SNAI1 and PAI-1. Firstly, there is a strong correlation between SNAI1 expression and metastatic process [18,19,68-70], whereas, SNAI1 repression decreases invasive behaviour [17,55]. Secondly, PAI-1 expression is correlated with adverse patient outcome and metastatic disease [35,38], and is down-regulated in our study after SNAI1 functional blockade.

Thus we suggest that either through E-cadherin expression and NFkB regulation or through binding to the E-box motif, SNAI1 is able to influence PAI-1 expression. This highlights the pivotal role of SNAI1 in cancer cell migration, influencing PAI-1, a key factor in metastatic cell escape. Therefore, we focused our attention on this possible link between SNAI1 and PAI-1 in the dynamic of migration and morphology of cancer cells.

### Quantitatively and qualitatively, the migration of SNAI1-DN cells is modified

**Figure 3:**
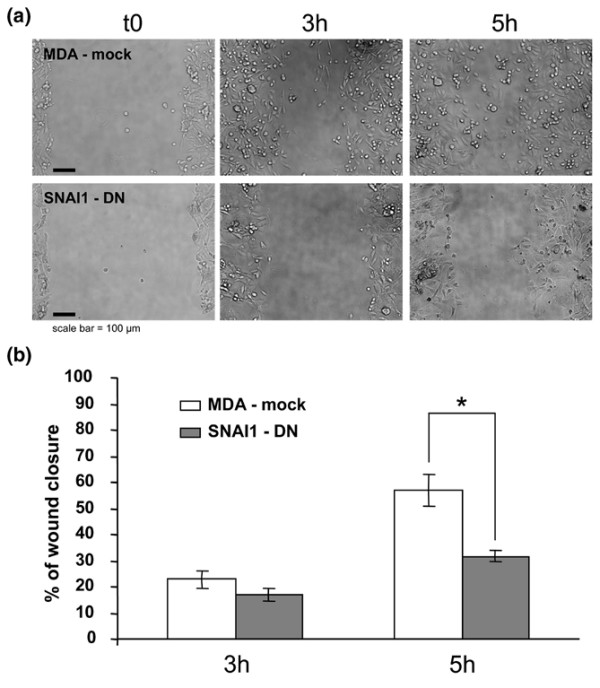
**Quantitative changes in SNAI1-DN cell migration**. The migratory behaviour of MDA-mock and SNAI1-DN cells is analysed in an *in vitro *wound healing assay. Monolayer cell cultures of MDA-mock and SNAI1-DN cells are gently scratched with a pipette tip to produce a wound. (a) Photographs of the cultures are taken immediately after the incision, and after three and five hours in culture. (b) The extent of wound closure is determined after three and five hours incubation. The comparison of MDA-mock and SNAI1-DN migration shows that SNAI1-DN cells migrate significantly more slowly than MDA-mock cells five hours after wounding. Mean values (standard error of the mean) of four independent assays are shown.* p < 0.05.

We performed an *in vitro *wound-healing assay to assess the consequences of SNAI1 blockade. The migration of MDA-mock and SNAI1-DN cells differs at three and five hours after wounding with SNAI1-DN cells migrating almost twice as slowly as MDA-mock cells (Figure 3a, b). These results are consistent with the prognostic value of SNAI1 in tumoural disease and metastatic potential [8,71,72].

**Figure 4:**
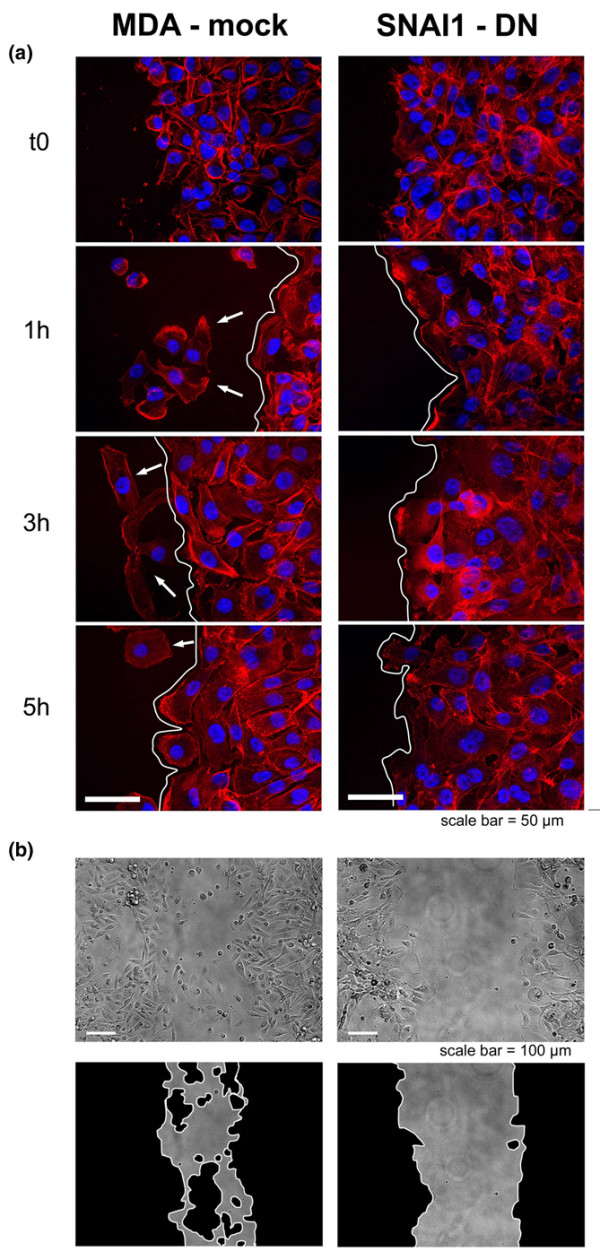
**Qualitative changes in SNAI1-DN cell migration**. In a wound healing assay, cells are fixed one, three and five hours after wounding. Together with the individual versus collective migration, morphological characteristics reinforce the epithelial to mesenchymal transition (EMT) status of MDA-mock and inverse transition for SNAI1-DN cells. (a) Actin immunostaining show that the MDA-mock cells move as individuals or as small groups of cells. The remaining population is not organised as a coherent group but rather as a juxtaposition of individuals. This is attested to by the presence of small groups of cells or individuals (white arrows) distinct from the leading edge (white line). In contrast, SNAI1-DN cells, moved as a coherent group, with a unique leading edge and large lamellipodia underlined by actin staining (see also figure 5). (b) This picture (at four times lower magnification) generalises the individual versus collective behaviour of the MDA-mock and SNAI1-DN cells at the wound scale.

The cell populations of the two cell lines migrate differently in terms of velocity (Figure 3). F-actin staining of the cells bordering the injury site also show morphological modifications of the cell migration (Figure 4a). We observed that although parental cells migrate primarily individually (i.e. as little groups of cells or as individuals), SNAI1 blockade resulted in a more collective migration phenotype. The control population (mock) appears not to be organised as a coherent group but rather as a collection of individuals. This is demonstrated by the absence of a unique leading edge, shown by actin cytoskeleton staining. In contrast, the SNAI1-DN cells, appeared to move as a coherent group, with a unique leading edge (Figure 4a). This is even more apparent at the lower magnification (Figure 4b).

In addition to examining morphology of the mock and SNAI1-DN cell lines, in wound healing assays, we noticed, as other groups have [73], that individual migration is associated with low E-cadherin expression while collective migration is associated with re-expression of E-cadherin.

### During wound healing, SNAI1-DN cells display a homogeneous leading edge with lamellipodia

We characterised the morphology of the two cell lines, because it is now well known that EMT can lead to the modification of cell morphology and individual migration. In collective migration, cellular groups migrate via a sequence of adhesion/traction at the leading edge implicating actin-mediated ruffling, invadopodia, integrin-mediated adhesion and recruitment of surface proteases [57].

In the present study, higher magnification and F-actin staining of the cells bordering the injury site (Figure 4a) show differences in the cell morphology of SNAI1-DN cells and MDA-mock cells. SNAI1-DN cells moved as a coherent group with a unique leading edge and numerous large lamellipodia underlined by actin staining and this migration was associated with a homogeneous direction towards the wound (Figure 4a). In contrast, in MDA-mock cells no lamellipodia and no similar directionality could be observed.

### PAI-1 distribution is modified in SNAI1-DN cells

**Figure 5:**
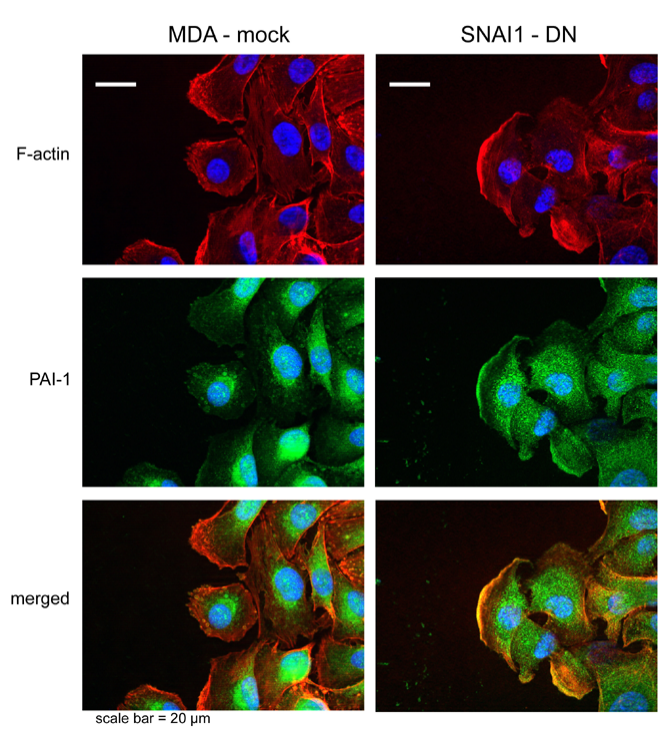
**Modified distribution of Plasminogen Activator Inhibitor type-1 (PAI-1) in SNAI1-DN cells**. PAI-1 immunostaining is rarely co-localied with F-actin structures in MDA-mock cells. In these cells, PAI-1 is preferentially localised in the perinuclear region. In contrast in SNAI1-DN-cells, PAI-1 immunostaining is strongly localised in the numerous lamellipodia and is more diffuse in the cytoplasm.

PAI-1 immunostaining is rarely colocalised with F-actin structures in MDA-mock cells. In these cells, PAI-1 is preferentially localised in the perinuclear region (Figure 5, MDA-mock). In contrast, in SNAI1-DN cells not only is the nuclear area decorated with PAI-1 antibodies, but so is the cell membrane and particularly the lamellipodia (Figure 5, SNAI1-DN). It is interesting to observe that in this cellular structure, PAI-1 and actin are co-localised (Figure 5, SNAI1-DN, 'merged'). It has previously been observed that PAI-1 is produced after injury by cells immediately adjacent to the wounding edge [74,75]. Providence and colleagues [75] demonstrated that *in vitro *wounding resulted in the identification of a 'leading edge' cohort (i.e. cells immediately adjacent to the wound border) becoming polarised with extended membrane ruffles and lamellipodia along the cellular 'face' juxtaposed to the denuded zone. These leading-edge cells were particularly immunoreactive with PAI-1 antibodies. Nevertheless, none of these studies demonstrate a PAI-1 distribution in lamellipodia associated with actin.

Taken together, the collective behaviour of cell migration, the numerous large lamellipodia at the leading edge and the co-localisation of actin with PAI-1 in lamellipodia, suggest that SNAI1 functional blockade redirected the cancer cells toward an epithelial-like cell behaviour. This behaviour is characterised by a lower expression of PAI-1 and altered distribution of PAI-1 protein in the cell, especially during wound healing. Likewise, the increased expression of E-cadherin mRNA suggests a partial recovery of epithelial properties in SNAI-DN cells. This morphological epithelial behaviour could represent an early step of the EMT reversion. Several studies have identified cells displaying both epithelial and mesenchymal traits. These 'metastable' cells present both residual E-cadherin and collective migration, membrane extension [76].

Finally, as pointed out by Providence and colleagues [77], the changes of PAI-1 expression and/or distribution to the cellular under surface that we previously described may be of importance, because PAI-1 would then come in close contact to focal contact sites [78]. These sites include integrin/vitronectin–dependent contacts that may be modified by the level of PAI-1 competing with integrins binding to vitronectin [33,41,43,79-81]. Thus PAI-1 may induce variations of cell attachment or detachment, especially in lamellipodia [43,82]. It has been shown that PAI-1 through co-ordinated interactions with plasminogen activators and the endocytic receptor LRP can control sequences of attachment/detachment in non-tumour cells [45,46]. This is consistent with our data with tumour cells presented here and suggests that in a similar way PAI-1 could promote cancer metastasis rather than inhibiting this process as predicted from studies of uPA. This could be of particular importance as cell matrix detachment is provoked by EMT inducers. The increase or decrease of PAI-1 expression is probably only one side of the story.

Focusing on the molecular and functional links between migration, E-cadherin, SNAI1 and PAI-1 we observed that elements inducing SNAI1 transcription, such as transforming growth factor beta, lead to the over-expression of PAI-1 and a decrease in E-cadherin with the subsequent situation being favourable to cell migration. In our study, the functional blockade of SNAI1 leads to reduced migration and redistribution of PAI 1 stressing the tight inter-connections between cells and their microenvironment.

In this regard, it may be important to highlight the differences between soluble PAI-1 playing the unique role of uPA inhibitor (i.e. decreasing proteolysis) and matrix bound PAI-1, which is mainly a matricellular protein (linking the cell membrane to the matrix) involved in cell adhesion/de-adhesion and migration [30,45,46].

Our study suggests that SNAI1 regulates PAI-1 expression, which is used as a matrix-bound regulator of cell migration and/or as a soluble inhibitor of proteolysis, according to SNAI1 function. Thus, SNAI1 [6] and PAI-1 [28-31,33,34] may influence cancer cell migration but, depending on SNAI1 status, this could imply proteolysis-dependent migration, collective or individual behaviour, and their consequences.

A better knowledge of the molecular requirements for EMT in human cancer will lead to a better understanding of tumour progression and to define more effective strategies for future therapeutic interventions.

## Conclusion

Both the transcription factor SNAI1 and the PA system influence cancer cell migration and are over-expressed in cancers. In this study we aimed to determine whether SNAI1 activity is correlated with expression of PA system components, and how this correlation can influence tumoural cell migration. Therefore, we compared the invasive breast cancer cell line MDA-MB-231 expressing Snail (MDA-mock) with its derived clone expressing a dominant-negative form of SNAI1 (SNAI1-DN).

We demonstrated by both cDNA microarrays and real-time quantitative RT-PCR that SNAI1-DN clone presents a significant decrease of PAI-1 and uPA mRNA compared with MDA-mock. With an *in vitro *wound healing assay, we observed that SNAI1-DN cells migrate more slowly than MDA-mock cells and in a more collective pathway. The blockade of SNAI1 activity resulted in the redistribution of PAI-1 in SNAI1-DN cells decorating large lamellipodia.

Taken together these results suggest that SNAI1 functional blockade is leading to partial re-expression of E-cadherin (i.e. at the level of transcription), to a decrease in PAI-1 and to a more collective migration, while the parental cells expressing SNAI1 have less E-cadherin, more PAI 1, and migrate individually. We suggest that the present study establishes a relation between SNAI1 function, PAI-1 distribution and EMT status.

Therefore, we believe that our results could lead to a better understanding of the permissive conditions for metastatic escape of cancer cells.

## Abbreviations

BSA: bovine serum albumin; C_T_: threshold cycle; DAPI: 4', 6-diamidino-2-phenylindole; ECM: extracellular matrix; EMT: epithelial to mesenchymal transition; FCS: fetal calf serum; MAT: mesenchymal to amoeboid transition; MDCK: Madin Darby Canin Kidney; MET: mesenchymal to epithelial transition; LRP: lipoprotein receptor-related protein; PA system: Plasminogen Activation system; PAI-1: Plasminogen Activator Inhibitor type-1; PBS: phosphate-buffered saline; uPA: urokinase Plasminogen Activator; uPAR: urokinase Plasminogen Activator Receptor; RT-PCR: reverse transcription – polymerase chain reaction.

## Competing interests

The authors declare that they have no competing interests.

## Authors' contributions

EF-G and MM contributed equally to this study. EF-G and CC-B performed RT-PCR, immunofluorescence analysis and migration assays. CC-B performed the statistical analysis. HP, AC, GM-B and JP participated in the construction of Snail-DN clone and performed microarray assays. MM with AC-M carried out the immunofluorescence analysis, migration assays and conducted imaging studies. BV was involved in cell cultures and in RT-PCR. EF-G, CC-B and MM participated in the wound healing assays. GBM and DL were involved in designing all experiments and writing the manuscript. All authors read and approved the final manuscript.

## Supplementary Material

Additional data file 1 A table that lists the human genes up-regulated or down-regulated in a clone expressing a dominant-negative form of SNAI1 (SNAI1-DN) compared with MDA-mock control cells. Negative sign (-) means down-regulation and positive sign (+) means up-regulation. Genes are organised by molecular function. A total of 99 genes were found to be differentially expressed, by at least a two-fold factor, in response to SNAI1 functional blockade. Click here for file
