# Supplementary material for: PAI-1 and functional blockade of SNAI1 in breast cancer cell migration
Source: Breast Cancer Res. 2008 Dec 3;10(6):R100. doi: 10.1186/bcr2203 (PMC2656896; doi:10.1186/bcr2203)
Supplement: Additional data file 1 — A table that lists the human genes up-regulated or down-regulated in a clone expressing a dominant-negative form of SNAI1 (SNAI1-DN) compared with MDA-mock control cells. Negative sign (-) means down-regulation and positive sign (+) means up-regulation. Genes are organised by molecular function. A total of 99 genes were found to be differentially expressed, by at least a two-fold factor, in response to SNAI1 functional blockade. [file bcr2203-S1.doc]

Supplementary Table: Human genes up- or down-regulated in a clone expressing a Dominant Negative form of SNAI1 (SNAI1-DN) compared to MDA-mock control cells. Negative sign (-) means down regulation and positive sign (+) means up regulation. Genes are organized by molecular function. A total of 99 genes were found to be differentially expressed, by at least a 2-fold factor, in response to SNAI1 functional blockade.

GenBank no. SNAI1l-DN Description

##### Cell cycle, proliferation and signalling (n=25)

| AA936768 | -8.82 | *IL1A,* interleukin 1, alpha |
| --- | --- | --- |
| AA401137 | -6.35 | *LCN2,* lipocalin 2 (oncogene 24p3) |
| AI074784 | -5.50 | *CSF3,*  colony stimulating factor 3 (granulocyte) |
| AA150507 | -4.97 | *IL1B,* interleukin 1, beta |
| AA994313 | -4.76 | *IL24,* ILERLUKINA 24 |
| N98591 | -4.49 | *IL6,* interleukin 6 (interferon, beta 2) |
| AA935273 | -4.28 | *GRO3,* GRO3 oncogene |
| R19956 | -3.97 | *VEGF,* vascular endothelial growth factor |
| W42723 | -3.84 | *GRO1,* GRO1 oncogene |
| AA417711 | -3.09 | *MAP4K4,* mitogen-activated protein kinase kinase kinase kinase 4 |
| H59620 | -2.48 | *INSIG1,* insulin induced gene 1 |
| H71474 | -2.43 | *FZD7,* frizzled (Drosophila) homolog 7 |
| AA454810 | -2.43 | *TACSTD2,* tumor-associated calcium signal transducer 2 |
| R80179 | 0.64 | *IL1R1,* interleukin 1 receptor, type I |
| AA233079 | 1.43 | *IGFBP1,* insulin-like growth factor binding protein 1 |
| AA777001 | 1.93 | *CCNA1,* cyclin A1 |
| AI340905 | 2.05 | *CCND3,* cyclin D3 |
| N72115 | 2.27 | *CDKN2C,* cyclin-dependent kinase inhibitor 2C (p18) |
| AA683550 | 2.29 | *IRAK1,* interleukin-1 receptor-associated kinase 1 |
| AA126947 | 2.35 | *CORO1C,* coronin, actin-binding protein, 1C |
| AA910443 | 2.78 | *NOV,* nephroblastoma overexpressed gene |
| AA489055 | 2.77 | *DHFR,* dihydrofolate reductase |
| AA873060 | 3.03 | *STMN1,* stathmin 1/oncoprotein 18 |
| H72122 | 3.06 | *ENC1,* ectodermal-neural cortex (with BTB-like domain) |
| R52796 | 11.28 | *IL13RA2,* interleukin 13 receptor, alpha 2 |

##### Cell death (n=7)

| R15728 | -6.24 | *GAS2,* growth arrest-specific 2 |
| --- | --- | --- |
| N94588 | -4.76 | *CFLAR,* CASP8 and FADD-like apoptosis regulator |
| H54629 | -3.96 | *TNFSF10,* tumor necrosis factor (ligand) superfamily, member 10 |
| R07870 | -2.51 | *BIRC3,* baculoviral IAP repeat-containing 3 |
| AI371096 | 2.08 | *DAPK1,* death-associated protein kinase 1 |
| AA444051 | 2.34 | *S100A10,* S100 calcium-binding protein A10 (annexin II ligand, calpactin I, light polypeptide (p11)) |
| AA488674 | 2.88 | *MCL1,* myeloid cell leukemia sequence 1 (BCL2-related) |

Immune response (n=3)

| g1846620 | -4.34 | *HLA-C,* major histocompatibility complex, class I, C |
| --- | --- | --- |
| g9888179 | -3.44 | *HLA-E,* major histocompatibility complex, class I, E |
| T62048 | -2.25 | *C1S,* complement component 1, s subcomponent |

Metabolism (n=10)

| AA448157 | -16.38 | *CYP1B1,* cytochrome P450, subfamily I (dioxin-inducible), polypeptide 1 (glaucoma 3, primary infantile) |
| --- | --- | --- |
| AA455235 | -7.74 | *ALDH1A3,* aldehyde dehydrogenase 1 family, member A3 |
| AA676466 | -3.27 | *ASS,* argininosuccinate synthetase |
| AA701963 | -3.27 | *AKR1B1,* aldo-keto reductase family 1, member B1 |
| N47312 | -3.16 | *HPRT1,* hypoxanthine phosphoribosyltransferase 1 (Lesch-Nyhan syndrome) |
| R63106 | -2.80 | *GSTM3,* glutathione S-transferase M3 (brain) |
| N66957 | -2.61 | *CYP27A1,* cytochrome P450, subfamily XXVIIA (steroid 27-hydroxylase, cerebrotendinous xanthomatosis), polypeptide 1 |
| AA983530 | -2.27 | *VNN1,* vanin 1 |
| H11036 | 0.62 | *PDP,* pyruvate dehydrogenase phosphatase |
| AA598652 | 1.76 | *SIAT1,* sialyltransferase 1 |

Transcription related (n=5)

| AA156988 | -2.97 | *ACO1,* aconitase 1, soluble |
| --- | --- | --- |
| AA664389 | -2.85 | *TSC22,* transforming growth factor beta-stimulated protein |
| AA973283 | -2.61 | *HDAC3* histone deacetylase 3 |
| W55872 | 0.53 | *NFKBIA,* nuclear factor of kappa light polypeptide gene enhancer in B-cells inhibitor, alpha |
| N66177 | 2.51 | *MITF,* microphthalmia-associated transcription factor |

Basic cellular function (n=13)

| AA429895 | -5.37 | *ABCC3,* ATP-binding cassette, sub-family C (CFTR/MRP), member 3 |
| --- | --- | --- |
| AA670429 | -3.27 | *SGNE1,* secretory granule, neuroendocrine protein 1 (7B2 protein) |
| N90204 | -2.88 | *UGCG,* UDP-glucose ceramide glucosyltransferase |
| AA453335 | -2.63 | *TXNRD1,* thioredoxin reductase 1 |
| R01732 | -2.52 | *AMPD3,* adenosine monophosphate deaminase (isoform E) |
| W77927 | -2.46 | *GBP2*, guanylate binding protein 2, interferon-inducible |
| H11320 | -2.42 | *UBA2,* SUMO-1 activating enzyme subunit 2 |
| R20379 | -2.31 | *EEF2,* eukaryotic translation elongation factor 2 |
| R49999 | -2.26 | *SLC19A1,* solute carrier family 19 (folate transporter), member 1 |
| N89671 | -2.22 | *RPL26,* ribosomal protein L26 |
| AA053165 | 0.64 | *PLEC1,* plectin 1, intermediate filament binding protein, 500kD |
| W86608 | 2.23 | *USP24,* ubiquitin specific protease 24 |
| AA872690 | 2.71 | *CCT6A,* chaperonin containing TCP1, subunit 6A (zeta 1) |

Miscellaneous (n=27)

| W78148 | -6.77 | *MGC5618,* hypothetical protein MGC5618 |
| --- | --- | --- |
| AA064973 | -4.57 | *CS-1,* calcineurin-binding protein calsarcin-1 |
| g4971953 | -4.36 | *EST,* Moderately similar to AF174605 1 F-box protein Fbx25 |
| AA046700 | -4.06 | *EST,* Weakly similar to AF174605 1 F-box protein Fbx25 |
| AA443832 | -3.18 | *LOC64182,* similar to rat myomegalin |
| R75819 | -2.90 | *FKBP2,* FK506-binding protein 2 (13kD) |
| R20669 | -2.56 | *TRA1,* tumor rejection antigen (gp96) 1 |
| W84445 | -2.40 | *RRAD,* Ras-related associated with diabetes |
| AA149579 | -2.34 | *PCANAP1,* prostate cancer associated protein 1 |
| AA005386 | -2.26 | *KIAA1915,* KIAA1915 protein |
| AA521414 | 0.52 | *CGI-85,* CGI-85 protein |
| AI669875 | 0.70 | *FAM8A1,* family with sequence similarity 8, member A1 |
| AA046724 | 1.47 | *SYNE-1B,* synaptic nuclei expressed gene 1b |
| R76614 | 1.61 | *NTN4,* netrin 4 |
| AA961735 | 2.03 | *MACMARCKS,* macrophage myristoylated alanine-rich C kinase substrate |
| W49781 | 2.17 | *LPXN,* leupaxin |
| AA036974 | 2.33 | *AOC3,* amine oxidase, copper containing 3 |
| H11732 | 2.36 | *CLECSF2,* C-type (calcium dependent, carbohydrate-recognition domain) lectin, superfamily member 2 (activation-induced) |
| AA598974 | 2.42 | *FLJ14365*, Homo sapiens cDNA FLJ14365 |
| AA934904 | 2.48 | *MGC5528,* hypothetical protein MGC5528 |
| AA131909 | 2.60 | *FLJ10540,* hypothetical protein FLJ10540 |
| AA426113 | 2.60 | *MGC4399,* hypothetical protein MGC4399 |
| W68220 | 2.68 | *KIAA0101,* KIAA0101 gene product |
| g9887904 | 2.87 | *NTRK1,* neurotrophic tyrosine kinase, receptor, type 1 |
| W73144 | 2.91 | *LCP1*, lymphocyte cytosolic protein 1 (L-plastin) |
| R45255 | 3.00 | *NOBP,* nuclear FGF3 binding protein |
| R38966 | 3.69 | *KIAA0462,* KIAA0462 protein |
